# Commentary on “The preferred surgical choice for intermediate-risk papillary thyroid cancer: total thyroidectomy or lobectomy? a systematic review and meta-analysis”

**DOI:** 10.1097/JS9.0000000000001705

**Published:** 2024-05-29

**Authors:** Abel Belay Mitiku, Eddie Bakiika, Mahalaqua Nazli Khatib, Sarvesh Rustagi, Prakasini Satapathy, Quazi Syed Zahiruddin, Ayush Anand

**Affiliations:** a Bishoftu Referal Hospital, Bishoftu, Ethiopia; b Makerere University College of Health Sciences, Kampala, Uganda; c Division of Evidence Synthesis; d South Asia Infant Feeding Research Network (SAIFRN), Division of Evidence Synthesis, Global Consortium of Public Health and Research, Datta Meghe Institute of Higher Education, Wardha; e School of Applied and Life Sciences, Uttaranchal University, Dehradun, Uttarakhand; f Center for Global Health Research, Saveetha Medical College and Hospital, Saveetha Institute of Medical and Technical Sciences, Saveetha University, Chennai; g MediSurg Research, Darbhanga, India; h Medical Laboratories Techniques Department, AL-Mustaqbal University, Hillah, Babil, Iraq; i BP Koirala Institute of Health Sciences, Dharan, Nepal

*Dear Editor*,

The management of intermediate-risk papillary thyroid carcinoma (IR-PTC) remains a contentious issue in thyroid surgery. While total thyroidectomy (TT) has traditionally been the preferred approach, recent evidence suggests that lobectomy (LT) might be an equally effective alternative with fewer complications. The systematic review and meta-analysis conducted by Cao *et al*.^1^ provide valuable insights into this debate, examining outcomes of TT and LT in IR-PTC patients.

The meta-analysis included eight retrospective cohort studies with a total of 2984 patients, making it one of the most comprehensive reviews on this topic to date^1^. The primary outcomes assessed were survival rates [overall survival (OS), disease-specific survival (DSS), recurrence-free survival (RFS)], recurrence rates, and postoperative complications. The study found no significant difference in OS, DSS, recurrence rates, and permanent postoperative complications between TT and LT. Both TT and LT showed comparable RFS rates [risk ratio (RR), 1.00; 95% CI, 0.96–1.05, *P* = 0.86]^1^. However, TT was associated with a higher incidence of transient complications, particularly transient parathyroid dysfunction (RR, 0.04; 95% CI, 0.01–0.15, *P* < 0.01)^1^.

The findings of this meta-analysis have significant implications for surgical practice in the treatment of IR-PTC. First, the comparable survival and recurrence outcomes between TT and LT suggest that LT might be sufficient for managing IR-PTC. This aligns with the principle of “less is more,” advocating for less extensive surgery, when possible, to reduce patient morbidity. Second, the higher incidence of transient complications associated with TT underscores the need to balance the extent of surgery with the risk of postoperative morbidity. LT, being less invasive, is associated with fewer transient complications, particularly transient hypoparathyroidism. Third, considering the similar efficacy in oncological outcomes, LT offers the advantage of preserving more of the thyroid function, potentially reducing the need for lifelong thyroid hormone replacement therapy, and thereby enhancing the patient’s quality of life.

While the study provides robust evidence supporting LT as a viable option for IR-PTC, several limitations must be acknowledged. First, all the included studies are retrospective cohort studies. Retrospective designs are susceptible to biases such as selection bias and information bias, which could affect the reliability of the findings. Second, the meta-analysis reports substantial heterogeneity in some subgroup analyses (e.g. OS and DSS). This variability could stem from differences in study design, patient populations, surgical techniques, and follow-up durations across the included studies. Third, the analysis includes only eight studies, which may limit the generalizability of the findings. A larger number of high-quality studies would provide more definitive conclusions. Fourth, the impact of adjuvant therapies, such as radioactive iodine treatment and thyroid hormone suppression therapy, was not consistently accounted for across the included studies. Variations in these treatments could confound the results and affect the comparability of outcomes. Fifth, although funnel plots were used to assess publication bias, the limited number of included studies may reduce the power of these assessments. Publication bias remains a concern and could influence the overall findings.

To address the limitations of the current evidence and provide more definitive guidance on the optimal surgical approach for IR-PTC, future research should focus on the following areas. First, high-quality randomized controlled trials (RCTs) are needed to compare TT and LT directly. Although challenging due to the need for large sample sizes and long follow-up periods, RCTs would provide the highest level of evidence. Second, in the absence of RCTs, well-designed prospective cohort studies with rigorous methodology and standardized data collection could offer valuable insights. Third, future studies should focus on long-term outcomes, including survival, recurrence, and quality of life, to fully understand the implications of each surgical approach. Fourth, research should systematically account for the use of adjuvant therapies and their impact on outcomes to isolate the effects of surgical approaches more accurately. Fifth, studies should incorporate patient-reported outcomes, such as quality of life, satisfaction, and functional status, to better understand the holistic impact of TT and LT on patients.

The meta-analysis by Cao and colleagues provides compelling evidence that lobectomy may be a preferable surgical option for patients with intermediate-risk papillary thyroid carcinoma. With comparable survival and recurrence outcomes to total thyroidectomy and a lower incidence of transient complications, lobectomy offers a less invasive yet effective approach. However, the limitations of the current evidence highlight the need for further research, particularly through high-quality RCTs and prospective studies, to confirm these findings and guide clinical decision-making.

## Ethical approval

Not applicable.

## Consent

Not applicable.

## Source of funding

Not applicable.

## Author contribution

A.B.M.: conceptualization, project administration, supervision, validation, writing—original draft and writing—review and editing. E.B.: conceptualization, project administration, supervision, validation, writing—original draft and writing—review and editing. M.N.K.: writing—original draft and writing—review and editing. S.R.: writing—original draft and writing—review and editing. P.S.: writing—original draft and writing—review and editing. Q.S.Z.: writing—original draft and writing—review and editing. A.A.: supervision, validation, and writing—review and editing.

## Conflicts of interest disclosure

The authors declare no conflicts of interest.

## Research registration unique identifying number (UIN)

Not applicable.

## Guarantor

Abel Belay Mitiku and Eddie Bakiika.

## Data availability statement

Not applicable.

## Provenance and peer review

Not commissioned, externally peer-reviewed
